# Molecular and morphological characterization of *Avenae*-group cyst nematodes (Heteroderidae) from Greece

**DOI:** 10.2478/jofnem-2025-0008

**Published:** 2025-03-19

**Authors:** Andrea M. Skantar, Zafar A. Handoo, Maria N. Hult, Alemayehu Habteweld, Maria Kormpi, Emannuel A. Tzortzakakis

**Affiliations:** Mycology and Nematology Genetic Diversity and Biology Laboratory, USDA-ARS-NEA-BA, Beltsville, MD 20705; Benaki Phytopathological Institute, Kifisia, Athens, Greece; Institute of Olive Tree, Subtropical Crops and Viticulture, Department of Viticulture, Vegetable Crops, Floriculture and Plant Protection, ELGO-DIMITRA, Heraklion, Crete, Greece

**Keywords:** *Heterodera filipjevi*, *H. hordecalis*, *H. mani*, Hsp90, mtCOI, ribosomal DNA, taxonomy

## Abstract

Cyst nematodes of the genus *Heterodera* comprise 87 nominal species of economically important plant parasites, with the *Avenae-*group one of the largest, consisting of 12 species. Samplings for cyst nematode studies were carried out from multiple locations in Greece from 2013 to 2021. Cysts of the genus *Heterodera* were recovered from potato fields, athletic stadium turfgrass and a garlic field. The recovered populations were identified using sequences of 28S, ITS1 and ITS2 rRNA, mitochondrial COI, and nuclear Hsp90. Using integrative taxonomic approaches, the recovered isolates were identified as *H. filipjevi* (from potato fields and turfgrass), *H. hordecalis* (from potato fields) and *H. mani* (from a garlic field), representing new records for Greece. Population diversity within each species was investigated using statistical parsimony of ITS rRNA and mtCOI, revealing haplotypes of the Greek populations and their relationships to others found in the Mediterranean basin and worldwide.

Cyst nematodes are serious parasites of many crops, with the genus *Heterodera* Schmidt, 1871 comprised of 87 species (Mulvey and Golden, 1983; Handoo and Subbotin, 2018; Li et al., 2020; Singh et al., 2020). Species of *Heterodera* can be divided into nine major groupings, including: *Afenestrata, Avenae, Bifenestrata, Cardiolata, Cyperi, Schachtii, Sacchari, Goettingiana,* and *Humuli* (Handoo and Subbotin, 2018). The taxonomy of the *Avenae-*group has been advanced by several review papers, as summarized in Handoo (2002), which also contains a key and compendium with morphometric and related details of all the life stages to facilitate identification. Systematics of the *Avenae* group was subsequently updated according to morphological and molecular traits (Subbotin et al., 2010; Handoo and Subbotin, 2018). It currently contains 12 species that are parasitic on monocotyledonous crops: *H. arenaria* Cooper, 1955; *H. avenae* Wollenweber, 1824; *H. aucklandica* Wouts and Sturhan, 1995; *H. australis* Subbotin, Rumpenhorst, Sturhan, and Moens, 2002; *H. filipjevi* (Madzhidov, 1981) Stelter, 1984; *H. hordecalis* Andersson, 1975; *H. latipons* Franklin, 1969, 1984, *H. mani* Matthews, 1971; *H. pratensis* Gäbler, Sturhan, Subbotin and Rumpenhorst, 2000; *H. riparia* (Kazachenko, 1993) Subbotin, Sturhan, Rumpenhorst and Moens, 2003; *H. sturhani* Subbotin, 2015; and *H. ustinovi* Kirjanova, 1969.

Characterization of the *Avenae* group was advanced through several biochemical and molecular studies (Ferris et al., 1994; Bekal et al., 1997; Subbotin et al., 1998, 1999, 2010). In recent years, *Heterodera avenae* group species have been studied through phylogenetic analyses of 28S and ITS ribosomal DNA and mitochondrial mtCOI barcodes (Subbotin et al., 2018; Mehalaine et al., 2020; Shao et al., 2023).

Despite the great economic importance of the damage to cultivated plants caused by *Heterodera* species, information regarding their occurrence in Greece is limited. The species *H. avenae*, *H. fici* Kir’yanova, 1954, and *H. schachtii* Schmidt, 1871 have been found on cereals, fig and sugarbeet, but their identifications were based only on morphological characteristics (Hirschmann et al., 1966; Koliopanos and Kalyviotis-Gazelas, 1969; Koliopanos and Vovlas, 1977; Kyrou, 1976; Kyrou, 1993). The presence of *latipons* and *H. humuli* in Greece was mentioned in two publications, but without morphological or molecular details (Greco et al., 2002; Akyazi et al., 2018), while *H. ripae* Subbotin, Sturhan, Waeyenberge et Moens, 2003 on nettle and *H. fici* were noted as originating from Greece in a publication related to molecular characterization of *Heterodera* spp. (Madani et al. 2004). Substantial efforts have been made in recent years to apply integrative taxonomic approaches to describe nematodes affecting various crops in Greece (Tzortzakakis et al., 2014, 2017, Palomares-Ruis et al., 2018; Archidona-Yuste et al., 2020; Clavero-Camacho et al., 2023). The only effort thus applied to *Heterodera* species from Greece was for *H. zeae* Koshy, Swarup & Sethi, 1971 on corn (Skantar et al., 2012).

The objectives of the present study were: *i*) morphological and morphometric characterization of *Heterodera* populations from Greece; *ii*) molecular characterization of the recovered populations using the internal transcribed space (ITS) and D2–D3 expansion segments of 28S rRNA, mitochondrial cytochrome oxidase I (mtCOI) DNA and partial nuclear heat shock protein 90 (Hsp90) gene sequences; and *iii*) haplotype diversity and intra- and interspecific variability of these populations in relation to existing molecular data for the species found.

## Materials and Methods

### Nematode species and populations

Soil samples collected from potato fields were investigated for presence of the potato cyst nematode (*Globodera* spp.) at the Nematology Laboratories of the Benaki Phytopathological Institute, Athens, Greece and of the Institute of Olive Tree, Subtropical Crops and Viticulture, Heraklion, Crete, Greece. Several cysts were observed and extracted from dried soil samples using a Fenwick can (Shepherd, 1986). Despite the presence or absence of *Globodera* spp., cysts of *Heterodera* species were found. Other soil samples investigated for the presence of nematodes came from a garlic field an athletic stadium turfgrass and processed for cyst extraction. Isolated cysts were preserved in DESS (Yoder et al., 2006) and were sent to the Mycology and Nematology Genetic Diversity and Biology Laboratory, USDA-ARS, Beltsville, MD, United States for further analysis. Other specimens used for molecular study were from −80°C frozen archival material preserved from Sicily, Italy and Oregon, U.S.

### Morphological analysis

Juveniles were fixed in 3% formaldehyde and processed to glycerin by the formalin glycerin method (Golden, 1990; Hooper, 1970). Females were fixed for 12 hr in 3% formaldehyde solution. Photomicrographs of cyst vulval cones, females, and J2 were made with an automatic 35-mm camera attached to a compound microscope having an interference contrast system. Photomicrographs of the specimens were made with a Nikon Eclipse Ni compound microscope using a Nikon DS-Ri2 camera. Measurements (Table 1) were made with an ocular micrometer on a Leica WILD MPS48 Leitz DMRB compound microscope and compared to the those from the original descriptions for each species (Matthews, 1971, Anderson, 1975; Madzhidov, 1981; Stelter, 1984). All measurements are in micrometers unless otherwise stated.

**Table 1:** Main morphometric characters of J2 from *Avenae*-group of *Heterodera* species recovered during present study from Greece including *H. filipjevi*, *H. hordecalis*, and *H. mani*. All measurements except lateral lines are in micrometers (µm) and in the form of mean (range).

**Morphometric characters**	***H. filipjevi***	***H. hordecalis***	***H. mani***
Body length	552 (462–662)	464 (417–525)	552 (526–578)
Stylet	25 (23–26)	24 (23–26)	25 (24–26)
Tail	61 (53–68)	57 (48–63)	65 (61–67)
Hyaline tail	41 (33–50)	36 (31–41)	41 (39–42)
Lateral lines	4	4	4

### Molecular methods

Single nematodes were mechanically disrupted with a scalpel in 20 μl nematode extraction buffer (500 mM KCl, 100 mM Tris-Cl (pH 8.3), 15 mM MgCl2, 10 mM dithiothreitol (DTT), 4.5% Tween 20 and 0.1% gelatin) and stored at −80°C until needed. Frozen nematodes were thawed, 1 μl proteinase K (from 2 mg/ml stock solution) was added, and the tubes were incubated at 60°C for 60 min, followed by 95°C for 15 min to deactivate the proteinase K. Two or three microliters of extract were used for each PCR reaction. Two to five individual nematodes for each population were used for molecular analysis. Markers examined were 28S and ITS rRNA, mtCOI, and nuclear Hsp90 genes, using primers and amplification conditions published previously (Table 2). Some ITS rRNA and all Hsp90 fragments were cloned with Strataclone PCR Cloning Kit (Agilent), purified with the Monarch Plasmid Miniprep Kit and sequenced by Genewiz, Inc. PCR products of 28S, mtCOI and ITS were purified with the Monarch DNA Gel Extraction Kit (NEB) and sequenced directly. Newly obtained sequences were submitted to GenBank with the accession numbers as listed in Table 3.

**Table 2:** Primers used for PCR amplification of DNA markers from *Heterodera* spp.

**Marker**	**Primers**	**Sequence 5′→3′**	**Primer and PCR References**
28S	D2Ab	ACAAGTACCGTGAGGGAAAGTTG	De Ley et al., 2005
	D3B	TCGGAAGGAACCAGCTACTA	Ye et al., 2007
ITS	TW81	GTTTCCGTAGGTGAACCTGC	Joyce et al., 1994
	AB28	ATATGCTTAAGTTCAGCGGGT	Skantar et al., 2012
mtCOI	Het-cox-1F	TAGTTGATCGTAATTTTAATGG	Subbotin, 2015
	Het-cox-1R	CCTAAAACATAATGAAAATGWGC	
Hsp90	U288	GAYACVGGVATYGGNATGACYAA	Skantar and Carta, 2005
	L1110	TCRCARTTVTCCATGATRAAVAC	

**Table 3:** GenBank accession numbers of DNA markers from *Avenae*-group species including *H. filipjevi H. hordecalis* and *H. mani* generated for this study.

**Species name**	**Code**	**Host**	**Location, year**	**GenBank accession numbers**			

				**28S**	**ITS**	**mtCOI**	**Hsp90**
*Heterodera filipjevi*	86G	Potato field	Viotia Greece, M1, 2013	PQ098459-PQ098461	PQ096808-PQ096810	PQ096970, PQ096971	PQ143935-PQ143941
	96E	Potato field	Livadia Greece, B12, 2015	PQ098464-PQ098467	PQ096802-PQ096804	PQ096975, PQ096976	PQ143942-PQ143945
	115H	Turfgrass	Athens, 2021	PQ098468-PQ098470	PQ096811-PQ096814	PQ096972-PQ096974	
	35A	Wheat	Oregon, USA, 2008	PQ098456-PQ098458	PQ096805-PQ096807	PQ096969, PQ096977	PQ143930-PQ143934
*Heterodera hordecalis*	86H1	Potato field	Laconia, Greece, M2, 2013	PQ098462-PQ098463	PQ096819-PQ096821	PQ096964-PQ096965	PQ143946-PQ143950
*Heterodera mani*	116A	Garlic field	Thiva, Greece, 2021	PQ098471-PQ098472	PQ096815-PQ096818	PQ096966-PQ096968	PQ143951-PQ143956

Multiple sequence alignments were made in Clustal W or MAFFT using default parameters within Geneious Prime 2024.0.7 and manually edited to remove poorly aligned regions at the ends or sequences of poor quality. For each marker, sequences from the same isolate were aligned with each other to determine intraspecific variation therein. Sequence matches in GenBank were determined by BLASTn analysis against the NCBI database. ITS, 28S D2–D3 rRNA, mtCOI, and nuclear Hsp90 sequences from the Greek isolates were separately aligned for each species with available *Heterodera* sequences from GenBank. Statistical parsimony networks were constructed from individual species sequence alignments using PopArt (Leigh and Bryant, 2015). Numbers listed on branches within the phylogenetic networks indicate the number of bp differences between haplotypes.

Phylogenetic trees were obtained for 28S D2–D3, ITS, and mtCOI datasets using Neighbour-Joining (NJ), maximum likelihood (RAxML) and Bayesian Inference (BI) within Geneious Prime or on the CIPRES Science Gateway (http://www.phylo.org; Miller et al., 2010). Outgroups for each marker were chosen based on previously published phylogenies. Best fitting models of nucleotide substitution were estimated using jModelTest2 (Darriba et al., 2012). BI analysis included a random starting tree and was run with four chains for 2 × 10^6^ generations. Two runs were performed for each analysis. Trees were sampled every 100^th^ generation, with 25% of results discarded as burn-in. Remaining samples were used to generate a 50% majority-rule consensus tree. Posterior probabilities (PP) are shown on appropriate clades.

## Results

Morphologically, the *Avenae* group is characterized by a lemon shape cyst, bifenestrate cone, short vulval slit, and the presence of bullae. Characteristics of second-stage juveniles including body, stylet, tail and hyaline region of tail length, stylet knobs nature, tail, hyaline tail terminus, and nature and number of lines in lateral field are also useful (Handoo, 2002).

### Greek population of *Heterodera filipjevi* (Madzhidov, 1981) Stelter, 1984

#### Figs 1,2; morphometric data in Table 1

##### Juveniles

**Figure 1:**
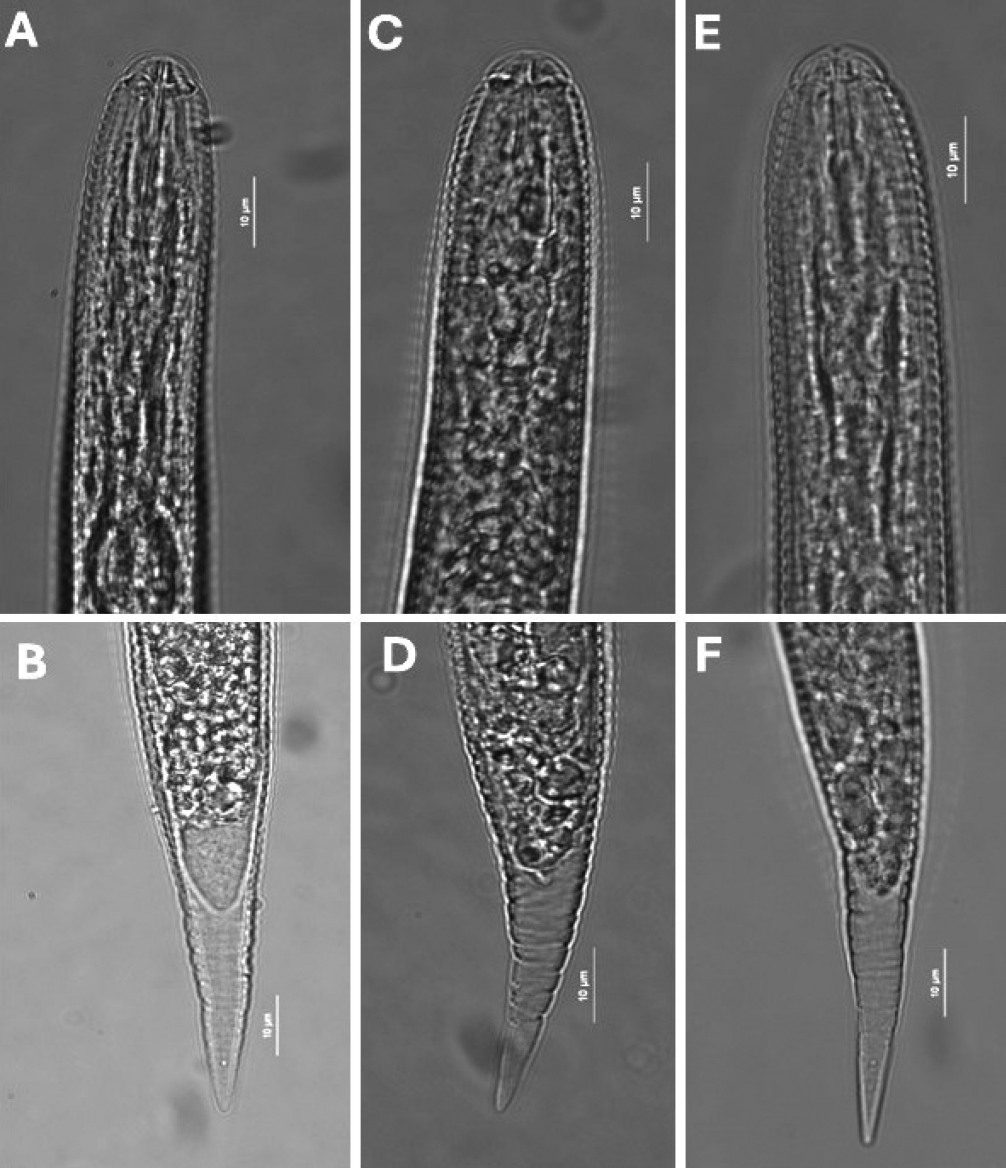
Photomicrographs of J2 showing lip region (top row) and tail regions (bottom) of *Heterodera* species (*Avenae* group) from Greece. (A, B) *H. filipjevi*; (C, D) *H. hordecalis*; (E, F) *H. mani*. Scale bar: 10 μm.

Lip region offset with two annuli. Stylet well developed with anchor-shaped basal knobs, tail conical with hyaline tail terminus (Fig. 1A, 1B). The lateral field with four lines of which the inner two are more distinct.

##### Cysts

**Figure 2:**
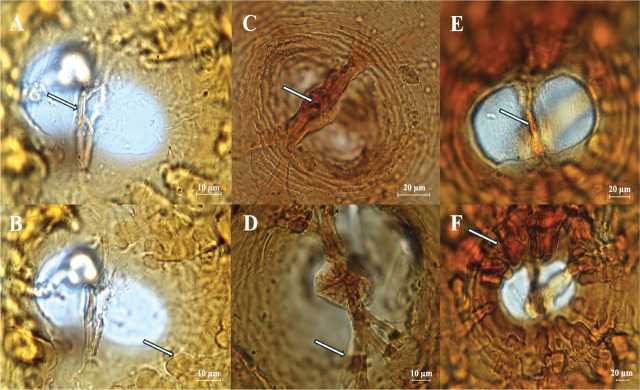
Photomicrographs of vulva cones of cyst showing vulva fenestra (all bifenstrate), vulva slit, bullae, and underbridge of *Heterodera* species, *Avenae* group. *Heterodera filipjevi*: (A) vulval slit (arrow) and underbridge; (B) Bullae (arrow); *H. hordecalis*: (C) vulval slit (arrow) and strong underbridge with bifurcate ends (arrow); (D) Bullae absent; *H. mani*: (E) vulval slit (arrow) and underbridge; (F) bullae (arrow). Images were captured at different focal planes to emphasize diagnostic features. Scale bars, A, B, D, 10μm; C, E, F, 20 μm.

Lemon-shaped and light brown colored. Bifenestrate. Cyst wall characteristic, thin with a zigzag pattern. Vulval cone bifenestrate with horseshoe-shaped semifenestra. The cysts were further characterized by heavy underbridge, short vulval slit, and many bullae (Fig. 2A, 2B).

#### Remark

Morphologically *H. filipjevi* differs from closely related species *H. avenae* by presence of an underbridge and from *H. ustinovi* by the shorter J2 tail (53–68 vs 80–94 µm) and shorter hyaline tail region (33–50 vs 52–62 µm). In addition, it differs from all *Avenae* group species by having a characteristic very thin cyst wall.

##### Molecular characterization

For *H. filipjevi*, the amplified fragments ranged in size from 711 to 755 bp for 28S, 889 to 1066 bp for ITS, 431 to 493 bp for mtCOI, and 1332 to 1342 bp for Hsp90. Differences in final sequence size were generally due to trimming of low-quality regions of sequences in 5′ and 3′. Intraspecific variation, accession numbers of the top matching sequences, degrees of sequence overlap, and corresponding percent similarities are shown in Table 4. Species identity was confirmed by BLASTn, showing the closest matches were to known *H. filipjevi* sequences, including 99.7–100% for 28S, 99.8–100% for ITS, and 98.3–99.8% for mtCOI. For Hsp90, the closest possible match was to *H. avenae* (86.9–87.8% identity) as the present study represents the first representatives of this gene obtained from *H. filipjevi*.

**Table 4:** Molecular similarity of 28S, ITS, mtCOI, and Hsp90 sequences obtained from Greek isolates of *Avenae* group species relative to existing sequences in GenBank. Intraspecific variation refers to differences among sequences recovered from each new population.

**Marker**	**Species name**	***Heterodera filipjevi***	***Heterodera filipjevi***	***Heterodera filipjevi***	***Heterodera filipjevi***	***Heterodera hordecalis***	***Heterodera mani***

	**Source**	**Potato field**	**Potato field**	**Turfgrass**	**Wheat**	**Potato field**	**Garlic field**
	Location, year	Viotia Greece, M1 2013	Livadia Greece, B12 2015	Athens, Greece 2021	Oregon, USA 2008	Laconia, Greece M2, 2013	Thiva, Greece 2021
**28S**	Population Code	86G	96E	115H	35A	86H	116A
	intraspecific variation^*^	0 bp	0 bp	0 bp	0 bp	0 bp	0 bp
	closest GenBank hit	GU083592	MG859980	MG859980	GU083592	LT159829	OQ918098
		*H. filipjevi*	*H. filipjevi*	*H. filipjevi*	*H. filipjevi*	*H. hordecalis*	*H. mani*
**ITS**	coverage	100%	100%	100%	99%	100%	98%
	% identity	100%	100%	100%	99.7%	100%	100%
	intraspecific variation^*^	0 bp	0–4 bp	1–6 bp	0 bp	0–1 bp	7–10 bp
	closest GenBank hit	MT254744	MT254744	MT254744	MT254744	MK840642	MG523157
		*H. filipjevi*	*H. filipjevi*	*H. filipjevi*	*H. filipjevi*	*H. hordecalis*	*H. mani*
**COI**	coverage	100%	100%	98%	100%	100%	100%
	% identity	100%	99.8%	99.9%	100%	99.0%	99.8%
	intraspecific variation^*^	0 bp	0 bp	0 bp	1 bp	2 bp	0 bp
	closest GenBank hit	MG523083	MK093059	MK093059	MK093059	MG5232140	MG523097
		*H. filipjevi*	*H. filipjevi*	*H. filipjevi*	*H. filipjevi*	*H. hordecalis*	*H. mani*
**Hsp90**	coverage	90%	96%	84%	97%	88%	99%
	% identity	99.5%	98.3%	99.0%	99.8%	91.48%	100%
	intraspecific variation^*^	3–64 bp	0–16	n/d	12–24 bp	4–9 bp	0–11 bp
	closest GenBank hit	MH848608	MH848608		MH848608	JQ316192	MH484608
		*H. avenae*	*H. avenae*		*H. avenae*	*H. avenae*	*H. avenae*
	coverage	93%	93%		92%	99%	99%
	% identity	87.4%	87.8%		86.9%	82.9%	92.6%

* Among sequences from the same population.

### Greek population of *Heterodera hordecalis* Anderson, 1975

#### Figs 1,2; morphometric data in Table 1

##### Juveniles

Lip region slightly offset (Fig. 1C) with three to four annules. Lateral field with four lines, outer lines areolated. Stylet with forwardly projecting knobs. Tail terminus finely rounded (Fig.1D).

##### Cysts

Ovoid with well-developed neck. Semifenestrate. Underbridge strong with bifurcate and branched edges. Vulval slit long, 17–23 µm. Bullae absent (Fig. 2C, D).

#### Remark

*Heterodera hordecalis* looks similar *H. latipons* but differs from it by longer vulval slit (17–23 vs 6–16 µm) and, more anteriorly projected stylet knobs in J2 than in *H. latipons*.

##### Molecular characterization

For *H. hordecalis,* the amplified PCR fragments ranged in size from 754 to 755 bp for 28S, 909 to 932 for ITS, 453 to 462 for mtCOI, and 1218 to 1219 for Hsp90. The BLASTn revealed ITS and mtCOI most matched those of other sequences of *H. hordecalis* populations (99% and 91.48%, respectively), although BLASTn of 28S and Hsp90 gave highest scores for *H. avenae* (100% and 82.95% identity, respectively), while they did not appear among the top hits due to shorter sequence overlap. Two 28S *H. hordecalis* sequences from Algeria (accession numbers LT159828 and LT159829) were 98.7 and 100% identical to the Greek sequences (9 and 0 bp differences, respectively). Hsp90 sequences most closely matched *H. avenae* (82.9% identity) because this study represents the first representatives of this gene obtained from *H. hordecalis*.

### Greek population of *Heterodera mani* Matthews, 1971

#### Figs 1,2; morphometric data in Table 1

##### Juveniles

Lip region with four indistinct annuli (Fig. 1E). Lateral field with four lines, outer lines indistinct and inner two ones clearly visible. Stylet well developed with characteristic well developed knobs with anterior ends deeply concave. Tail conoid, tapering uniformly to a finely rounded terminus.

##### Cysts

Spherical, dark brown to pale in color (Fig. 2E, F). Body wall with zigzag lines. Tick subcrystalline layer present. Bifenestrate. Underbridge weak or absent. Bullae present.

##### Molecular characterization

For *H. mani,* the amplified fragments ranged in size from 745 to 746 bp for 28S, 914 to 916 bp for ITS, 489 to 505 bp for mtCOI, and 1243 to 1249 bp for Hsp90. Sequences from the Greek isolate all closely matched those from previously reported *H. mani* sequences, with 100% to 28S, 99.8% to ITS, and 100% to mtCOI as shown in Table 4. Hsp90 sequences from this isolate all matched *H. avenae* (92.6% identity). There were no exact species matches for Hsp90 sequences because this study represents the first representatives of this gene obtained from *H. mani*.

### Haplotype networks

**Figure 3:**
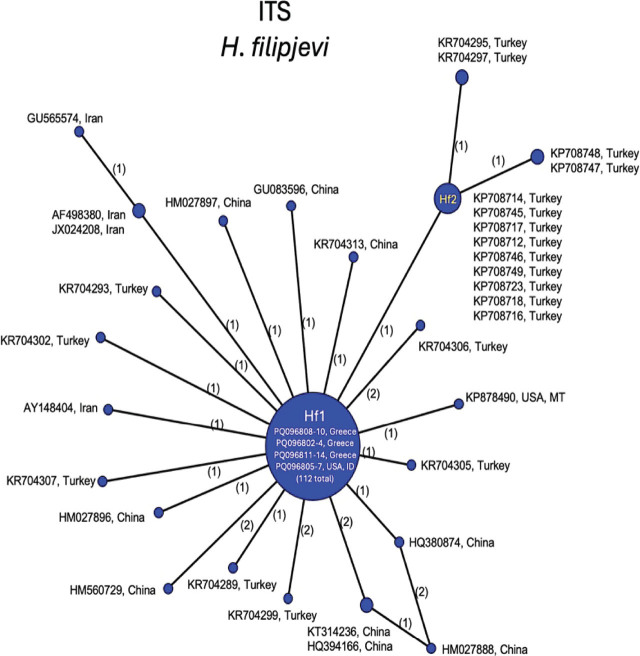
Statistical parsimony network showing phylogenetic relationships among ITS rRNA haplotypes of *H. filipjevi* populations. Circle size is proportional to the number of sequences with each haplotype. New sequences are in bold. Numbers on branches indicate bp differences between haplotypes.

Statistical parsimony analysis was used to highlight the ITS and mtCOI haplotype variation of *Avenae* group cyst nematode populations representing a wide range of geographic isolates, including the present populations from Greece. For *H. filipjevi,* the 509 bp ITS alignment included 146 sequences. There were two main haplotypes in the phylogenetic network (Fig. 3), a dominant one Hf1 shared by the present populations from Greece and the U.S., shared by 112 sequences in total, covering a wide geographic distribution (China, Germany, Iran, Italy, Kyrgyzstan, Russia, Slovakia, Spain, Tajikistan, Turkey, United Kingdom and the U.S.). Another dominant haplotype Hf2 was shared by populations from Turkey. The maximum intraspecific variation among *H. filipjevi* ITS was 1.6%.

**Figure 4:**
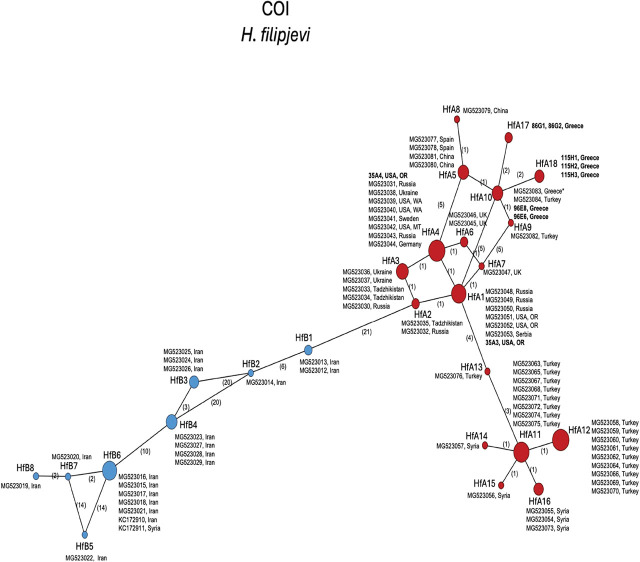
Statistical parsimony network showing phylogenetic relationships among mtCOI haplotypes of *H. filipjevi* populations. Circle size is proportional to the number of sequences with each haplotype. New sequences are in bold. Numbers on branches indicate bp differences between haplotypes. The sequence MG523083* in haplotype HfA10 was determined to have originated from the Livadia, Greece population but was mistakenly attributed to Crete.

The 415 bp mtCOI alignment of *H. filipjevi* included 84 sequences. For context, the mtCOI phylogenetic network (Fig. 4) followed the same haplotype naming scheme of Subbotin et al. (2018), including major groupings A (red circles) and B (blue circles). COI sequences from the Livadia, Greece population shared HfA10 haplotype with sequences from Turkey and another described as from Crete. The Viotia and Athens populations appear as new haplotypes here named HfA17 and HfA18, respectively. The U.S. (Oregon) sequences were split between HfA4, which included other U.S. isolates from Washington and Montana, as well as Sweden, Russia, Ukraine, and Germany, and HfA1, which included other sequences from Oregon, U.S. as well as Serbia and Russia. B types were from Iran and Syria. Maximal intraspecific variation was 10.6%.

**Figure 5:**
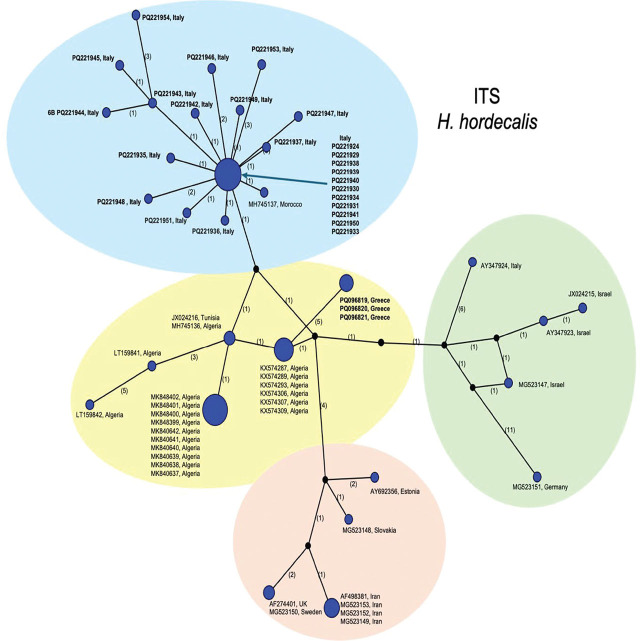
Statistical parsimony network showing phylogenetic relationships among ITS rRNA haplotypes of *H. hordecalis* populations. Circle size is proportional to the number of sequences with each haplotype. New sequences in bold.

The ITS alignment of *H. hordecalis* was 733 bp long and contained 63 sequences. The phylogenetic network (Fig 5) revealed the Greek isolate was most related to haplotypes common in Algeria and Tunisia (shaded in yellow), differing at 5–6 bp. Another common haplotype included multiple sequences from Italy and a population from Morocco (shaded in blue). A less common grouping included haplotypes from Iran, Sweden, the United Kingdom, Slovakia and Estonia (shaded in orange). Lastly, isolates from Israel grouped with isolates from Germany and Italy (shaded in green). Maximum intraspecific variation was 3.4%.

**Figure 6:**
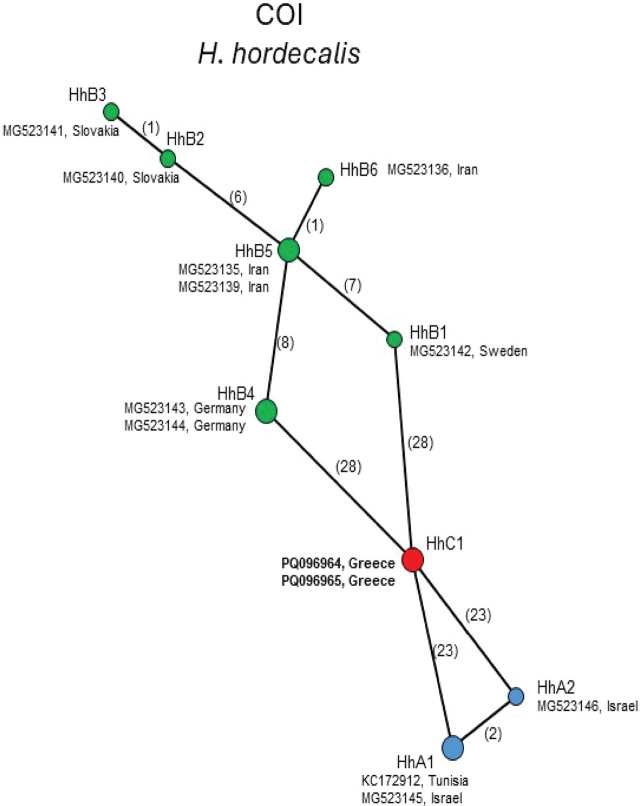
Statistical parsimony network showing phylogenetic relationships among mtCOI haplotypes of *H. hordecalis* populations.

The 405 bp mtCOI alignment of *H. hordecalis* included 13 sequences. The haplotype network shown in Fig. 6 follows the same naming scheme as in Subbotin et al. (2018), with the new Greek sequences (given the name haplotype HhC1, red circle) occurring midway between A types from Israel and Tunisia (23 bp differences) and B types from Sweden, and Germany (28 bp differences). Maximal overall intraspecific sequence diversity was 11.5%.

**Figure 7:**
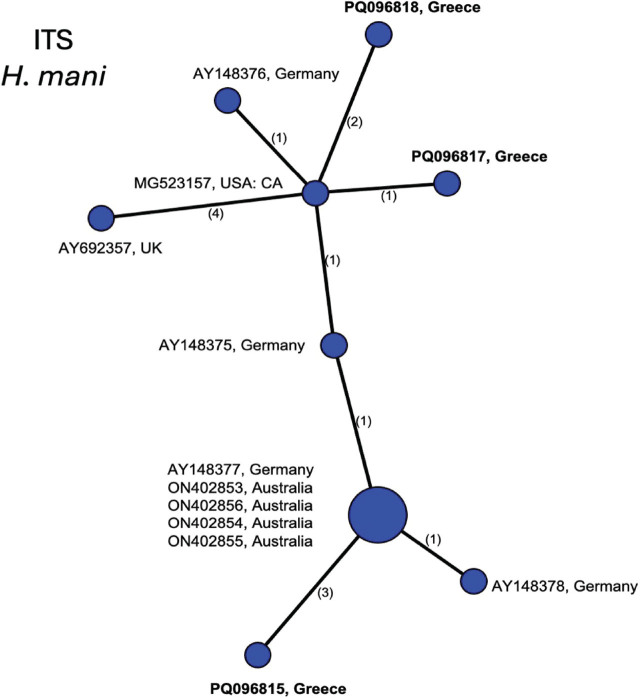
Statistical parsimony network showing phylogenetic relationships among ITS rRNA haplotypes of *H. mani* populations. Circle size is proportional to the number of sequences with each haplotype. New sequences in bold.

The *H. mani* ITS alignment was 930 bp and contained 13 sequences. The haplotype network is shown in Fig. 7. The most common haplotype consisted of sequences from Australia and Germany. The sequences from Greece were 1–4 bp different from those from the main haplotype or to those from the U.S. or Germany. The COI alignment contained 13 sequences of 346 bp. All except sequences from Australia which contained ambiguities were of an identical haplotype (network not shown).

### Phylogenetic trees

**Figure 8:**
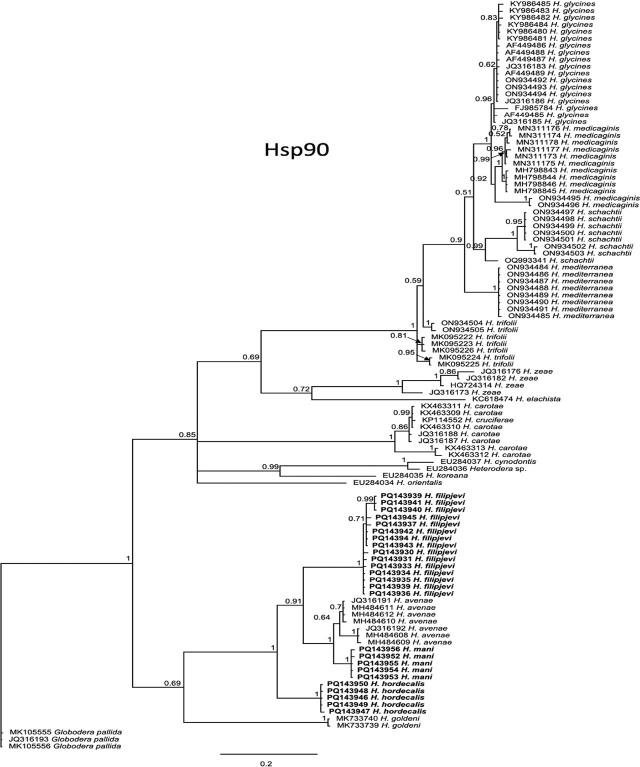
Phylogenetic relationships of *Heterodera Avenae* group species from Greece inferred from partial Hsp90 genomic DNA sequence alignments analyzed by Bayesian Inference. Posterior probabilities are shown on appropriate branches. New sequences in bold.

Phylogenetic trees inferred from 28S D2–D3, ITS, or COI contained no significant conflicts in branching order and support levels and conformed to *Heterodera* species trees previously published for these markers, so were not presented here. For Hsp90, phylogenetic trees were obtained by BI with the model of nucleotide substitution GTR + I + G according to Akaike information criterion and implemented within MrBayes (Huelsenbeck and Ronquist, 2001) using either a short DNA sequence alignment with more *Avenae* group taxa or long alignments with fewer taxa, respectively. One tree was inferred from an alignment of 519 bp that included 105 *Heterodera* sequences and three *Globodera pallida* outgroup sequences (Fig 8). Species represented only by short (~350 bp) Hsp90 sequences were not included in the tree based on the alignment of longer (~1 kb) sequences to avoid the presence of long gaps (tree not shown). Taxa common to both trees did not differ significantly in position or branch support. In both Hsp90 trees, *Avenae* group species formed well-separated, strongly supported clades (PP=99%), indicating that clear separation of species. Species in the *Schachtii* group (Fig. 8) were also separated into distinct, highly supported clades. Within the *Goettingiana* group *H. cruciferae*, Franklin, 1945 was not resolved from *H. carotae* Jones, 1950. Resolution of species in the other groups (*Afenestrata, Cardiolata, Cyperi, Humuli, Sacchari*) could not be determined from the limited number of sequences available.

## Discussion

This study characterized three *Avenae* group cyst nematodes reported for the first time in Greece, *viz*. *H. filipjevi, H. hordecalis,* and *H. mani*.

*Heterodera filipjevi* was first found by Kirjanova in 1941 in the Sverdlovsk region of Russia on wheat and considered as *H. avenae*. While this nematode was very similar in morphology to *H. avenae*, it differed from it by several morphometric characters and was therefore given the name *H. filipjevi* by Madzhidov (1981). It has since been found in association with 20 species of cereals and grasses (Popova, 1971; Bajaj and Kanwar, 2005) and is considered as an important pest of cereals worldwide (Hajihasani et al., 2010; Renco et al., 2018). Its distribution includes several countries in Europe, Asia, and North America (specifically, the U.S.). Within the Mediterranean region, *H. filipjevi* is known to occur in Italy, Spain, Syria, and Turkey (Subbotin et al., 2003; Madani et al., 2004; Abidou et al., 2005; Toumi et al., 2013). ITS sequences from the Greek populations shared the most common Hf1 haplotype with populations with broad geographic distribution. Mitochondrial COI sequences from Greece grouped most closely to those from Turkey, and other HfA haplotypes from China, Russia, the U.S., and Europe. The mtCOI contained greater intraspecific diversity than ITS for *H. filipjevi*. Haplotype groups were otherwise consistent with those determined previously (Subbotin et al., 2018). The current work comprises the first report of *H. filipjevi* from Greece.

The COI haplotype HfA10 of *H. filipjevi* included a sequence previously described as from Crete (Subbotin et al., 2018). Investigation of past records revealed that this sequence was generated using the same 2015 Livadia population from mainland Greece (via nematodes shared with those authors) but mistakenly attributed to Crete. B haplotypes were from Iran and Syria as shown previously (Subbotin et al. (2018).

*Heterodera hordecalis* was first described by Andersson (1975) on barley in Sweden. Within North America, it was reported in 1985 on Prince Edward Island, Canada, but this was based on invalid records (EPPO Global Database, 2019). It occurs on several other monocotyledonous hosts and has a wide distribution in countries of the Mediterranean region (France, Italy, Israel, Algeria, Morocco, and Tunisia) (Namouchi-Kachouri et al., 2005; Greco et al., 2002; Tirchi et al., 2016; Smaha et al., 2018; Subbotin et al., 2018). The Greek isolate of *H. hordecalis* was mostly related to populations from Algeria and Tunisia based on ITS (Tirchi et al., 2016), and the COI sequences occurred in an intermediate position between A and B haplotypes (Fig. 6). Greece was included in the countries of distribution of the species but there was no formal published report until now (Subbotin et al., 2010). Therefore, this work comprises the first characterization of *H. hordecalis* within the country. The ITS analysis included several unpublished *H. hordecalis* ITS sequences obtained from Sicilian populations. Available records are incomplete, but some of the populations were described previously but without molecular details (Lombardo et al., 2009, 2011). Unfortunately, further morphological and molecular analysis of those populations was not possible due to lack of specimens and supportive details. However, the ITS sequences add substantial context for *H. hordecalis* and expanded the maximum variation from 2.0% as reported by Subbotin et al. (2018) to 3.4% in the present study. COI from *H. hordecalis* contained greater intraspecific diversity than ITS.

*Heterodera filipjevi* and *H. hordecalis* have been reported as serious pests of cereal crops. However, damage to the potato (or turfgrass, in the case of the Athens isolate) was not assessed during the present study. Their presence in potato fields is likely attributable to previous cereal crops in the same fields since rotations of potato with cereals are quite common in the sampled areas. Further research is necessary to investigate whether they are pathogenic to other crops.

*Heterodera mani* was first described as a parasite on grasses in Northern Ireland, including perennial ryegrass *Lolium perenne* L. (Poaceae, Poales) designated as the type-host (Matthews, 1971). In the description of the nematode, two further hosts *Dactylis glomerata* L. and *Festuca pratensis* are mentioned, while in experimental tests infected ten further grasses (Mowat, 1974). *Heterodera mani* has been reported to occur in pastures and grasslands in Europe (Belgium, Estonia, France, Germany, Italy, The Netherlands, Slovakia, United Kingdom), South Africa, the U.S. (California, Washington) and in Western Australia and Tasmania (Subbotin et al., 2010; EPPO Global Database, 2018; Subbotin et al., 2018; Huston et al., 2023; Jain et al., 2023), although supportive molecular information is lacking for several of these reports. This is the first characterization of the species from Greece. *Heterodera mani* has not been reported previously on garlic. It is likely that the *H. mani* recovered from the garlic field was associated with grasses present as weeds between crops or from previous rotation crops. This finding further expands the known distribution of *H. mani* within Europe, but its impact on wild or cultivated grasses remains to be determined.

Phylogenetic trees of *Avenae* group species from BI analyses of 28S, ITS, or mtCOI DNA alignments were consistent with those previously published (Subbotin et al., 2018) in terms of overall tree topology and the expected placement of new *H. filipjevi, H. hordecalis,* and *H. mani* sequences, so were not presented here. Instead, the haplotype networks (Figs. 3–7) shed new light on the population genetic diversity within the *Avenae* group species examined and show that sequence diversity of mtCOI within *H. filipjevi* and *H. hordecalis* is generally higher than that observed for ITS. It has been shown previously that *H. mani* was only weakly separated from *H. avenae* based on only a few bp differences in ITS, but *H. mani* did form a separate clade within ITS phylogenetic trees (Huston et al., 2022; 2023). The *H. mani* mtCOI sequences from Greece, Australia, Germany, Tasmania, and the U.S. contained low intraspecific variation (0.2%) but showed remarkable differences from those of near-relative *H. pratensis* (27–29 bp, 8% differences). As additional populations are found, a better resolution of the molecular diversity should emerge.

Partial Hsp90 sequences have proved useful references for phylogenetic analysis of numerous cyst nematodes, including species of *Cactodera* Krall & Krall, 1978, *Globodera* Skarbilovich, 1959, *Punctodera* Mulvey & Stone, 1976, and the *Heterodera schachtii* group (Skantar et al., 2019; 2021; Kantor et al., 2020; Handoo et al., 2021; Hesse et al., 2021; Subbotin et al., 2024). In the present study, partial Hsp90 sequences representing approximately 50% of the full-length gene were obtained from several *Avenae* group isolates. Although Hsp90 was only available from a limited number of *Avenae* group species and populations, the tree reconstructed using this marker yielded in strongly supported clades. The sequences had sufficient variation for delineating *Avenae* group species. Within the *Goettingiana* group, the relation of *H. cruciferae* and *H. carotae* were not resolved by Hsp90, which was also the case ITS rRNA and COI (Escobar et al., 2018). Further analysis of this gene using additional populations and sequences of *Heterodera* species could help to achieve a better resolution of relationships unresolved by ribosomal and mitochondrial genes.
